# Nephrectomy for Metastatic Kidney Tumor in Patients with Differentiated Thyroid Cancer: A Report of Two Cases

**DOI:** 10.1155/2018/7842792

**Published:** 2018-11-11

**Authors:** Haruhiko Yamazaki, Takeshi Kishida, Go Noguchi, Hiroyuki Iwasaki, Nobuyasu Suganuma, Katsuhiko Masudo, Hirotaka Nakayama, Toshinari Yamashita, Takashi Yamanaka, Yuko Sugawara, Yuka Matsubara, Kaori Kohagura, Yasushi Rino, Munetaka Masuda

**Affiliations:** ^1^Department of Breast and Endocrine Surgery, Kanagawa Cancer Center, Yokohama, Japan; ^2^Department of Urology, Kanagawa Cancer Center, Yokohama, Japan; ^3^Department of Breast and Thyroid Surgery, Yokohama City University Medical Center, Yokohama, Japan; ^4^Department of Surgery, Yokohama City University School of Medicine, Yokohama, Japan

## Abstract

The occurrence of renal tumors originating from thyroid cancer is extremely rare with a few effective treatments for renal metastases. Here, we report the cases of two patients with differentiated thyroid cancer who underwent nephrectomy for a metastatic kidney tumor. Case 1 was a 74-year-old man who was diagnosed with right kidney tumor 10 years after initial surgery for papillary thyroid cancer (PTC). Right nephrectomy was performed, and the pathology was metastatic PTC. Case 2 was a 68-year-old woman who was diagnosed with left kidney tumor 24 years after surgery for follicular thyroid carcinoma (FTC). Left nephrectomy was performed, and the pathology was metastatic FTC. Nephrectomy for single renal metastasis could be considered a treatment option if the patients' general condition is positive.

## 1. Introduction

The occurrence of renal tumors originating from thyroid cancer is extremely rare with a prevalence of 0.47% [1]. Although radioactive iodine (RAI) therapy has been used to treat metastatic and recurrent differentiated thyroid cancer (DTC), no data establish the efficacy of RAI for the treatment of renal metastases. Thus, to date, effective treatment methods for renal metastasis from thyroid cancer remain unavailable. When a tumor is metastasized to the kidneys, it generally comprises other metastatic lesions or is bilateral [2]. Herein, we report cases of two patients with DTC who underwent nephrectomy for a metastatic kidney tumor.

## 2. Case Report

### 2.1. Case 1

**Figure 1:**
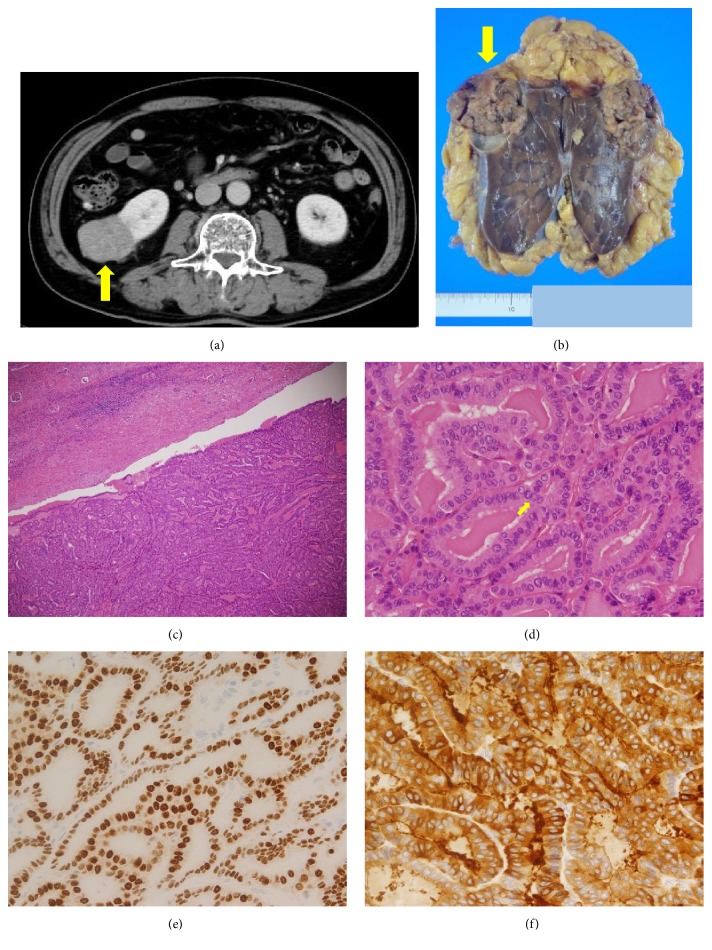
(a) Computed tomography showed the right kidney tumor (arrow). (b) The right nephrectomy specimen contained a grayish tumor measuring 5.5cm × 5.0 cm on the upper pole (arrow). (c, d) Histologic sections of the resection specimen showed that the tumor formed a papillary structure, and the lumen was filled with eosinophilic substances considered colloids. Individual cancer cells had nuclear grooves, and findings suggestive of nuclear inclusions were also observed (arrow). (c): Original magnification x200, (d): Original magnification x400. (e, f) Immunohistochemistry revealed positive for thyroid transcription factor 1 (e) and thyroglobulin (f). Original magnification x400.

A 74 -year-old man was diagnosed with right kidney tumor on routine computed tomography (CT) 10 years after initial surgery. His medical history comprised near total thyroidectomy for papillary thyroid cancer (PTC) 10 years ago and complete thyroidectomy for recurrence 6 years ago. He did not complain of urinary symptoms such as flank pain or hematuria. Blood test results were as follows: creatinine (Cre), 0.78 mg/dL; blood urea nitrogen (BUN), 14.2 mg/dL; thyroid-stimulating hormone (TSH), 0.13 *μ*IU/mL; free thyroxine (F-T4), 1.57 ng/mL; thyroglobulin (Tg), 95.0 ng/dL (Tg doubling time, 0.31 years); and Tg antibody (TgAb), 11 IU/mL. Transabdominal ultrasonography (US) revealed a right kidney tumor measuring 5.3 × 3.7 cm. The tumor blood flow was similar to that of the kidneys. In addition, CT revealed an irregular tumor mass projecting outward from the right kidney with no evidence of other metastatic lesions (Figure 1(a)). Despite a little marginally elevated Tg level, imaging studies of the right kidney raised suspicions of primary renal cell carcinoma (RCC). Following consultations with urologists, a right laparoscopic radical nephrectomy was performed. The pathology report revealed that the right nephrectomy specimen contained a grayish tumor measuring 5.5 × 5.0 cm on the upper pole (Figure 1(b)). Histological sections of the resected specimen revealed that the tumor formed a papillary structure, and the lumen was filled with eosinophilic substances that were considered colloids. Further, individual cancer cells had nuclear grooves, and findings suggestive of nuclear inclusions were observed (Figures 1(c) and 1(d)). Immunohistochemistry (IHC) results were positive for thyroid transcription factor 1 (TTF-1) and Tg (Figures 1(e) and 1(f)). The patient was discharged from the hospital on postoperative day 7 without any complications. Postoperatively, the Tg level decreased to 3.05 ng/dL and, 3 years after nephrectomy, no recurrence has been reported.

### 2.2. Case 2

**Figure 2:**
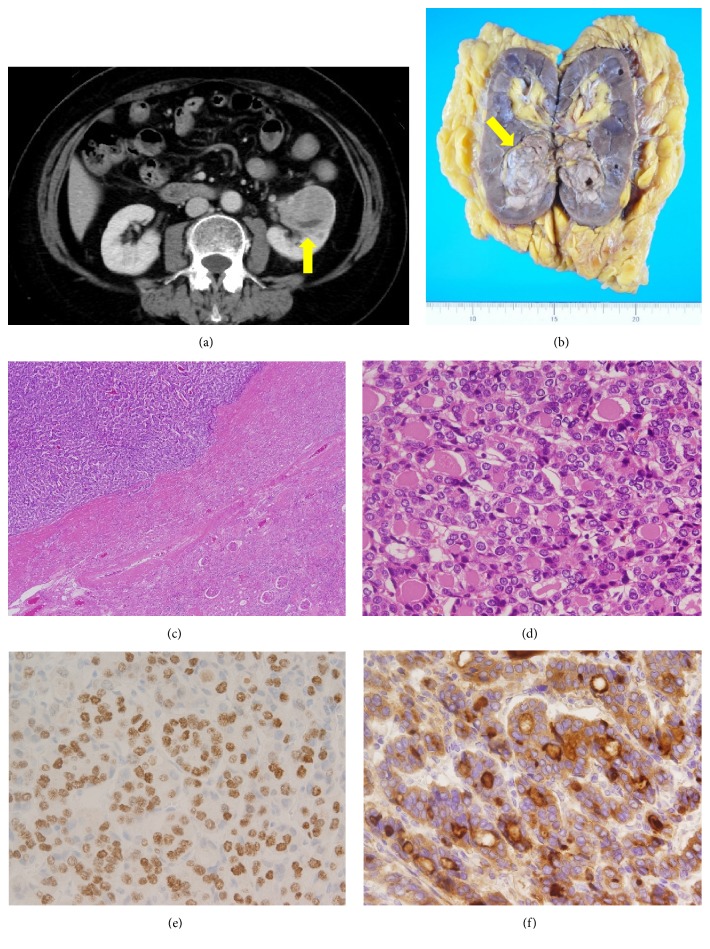
(a) Computed tomography showed the left kidney tumor (arrow). (b) The left nephrectomy specimen contained a light brown tumor measuring 4.5cm × 4.4 cm on the lower pole arrow. (c, d) Histologic sections of the resection specimen showed that the tumor formed a follicular structure, and was infiltrating and proliferating. (c): Original magnification x200, (d): Original magnification x400. (e, f) Immunohistochemistry revealed positive for thyroid transcription factor 1 (e) and thyroglobulin (f). Original magnification x400.

A 68-year-old woman, with medical history of total thyroidectomy for follicular thyroid carcinoma (FTC) 24 years ago, exhibited a high Tg level. However, she did not complain of any urinary symptoms. Her blood test results were as follows: Cre, 0.65 mg/dL; BUN, 14.7 mg/dL; TSH, 0.09 *μ*IU/mL; F-T4, 1.35 ng/mL; Tg, 10500.0 ng/dL (Tg doubling time, 0.31 years); and TgAb, 11 IU/mL. CT revealed a left kidney tumor measuring 4.0 × 3.5 cm (Figure 2(a)). The Tg level was remarkably high; thus, recurrence of FTC was predominantly suspected. However, CT identified no other metastatic lesion, and nephrectomy was performed. The pathology report revealed that the left nephrectomy specimen comprised a light brown tumor measuring 4.5 × 4.4 cm on the lower pole (Figure 2(b)). In addition, histological sections of the resected specimen revealed that the tumor formed a follicular structure and was undergoing infiltration and proliferation (Figures 2(c) and 2(d)). Furthermore, IHC was positive for TTF-1 and Tg (Figures 2(e) and 2(f)). The patient was discharged from the hospital on postoperative day 6 without any complications. The Tg level decreased postoperatively to 298 ng/dL.

## 3. Discussion

In 2012, approximately 230,000 and 70,000 new cases of thyroid cancer were estimated among females and males, respectively, with an age-standardized (world population) rate of 6.10/100,000 females and 1.90/100,000 males [3]. Both PTC and FTC are called DTC, accounting for >95% of all thyroid carcinoma cases [4]. The prognosis of DTC is good, and the disease-specific survival rate is reportedly >90% [5]. However, some DTCs develop distant metastasis, the frequency of which has been reported to be 4 %-15 %, with the lung being the most frequent metastatic site [6]. The origin of renal tumor originating from thyroid cancer is extremely rare, with a prevalence of 0.47% [1]. Frequently, metastatic renal tumors exhibit poor clinical symptoms, and patients die before developing symptoms. Thus, it is rare for the cancer to be discovered during survival and be treated [7]. The duration from the diagnosis of thyroid cancer to the detection of renal metastasis is long, and cases of >10 years have been reported previously [7, 8]. By the time renal metastasis was diagnosed in cases 1 and 2 of this report, 10 and 24 years had elapsed since initial surgery, respectively. In addition, both patients exhibited no subjective symptoms; however, kidney tumors were detected with whole body CT. The follow-up method following thyroid cancer surgery has recently changed from whole body RAI scanning to US and serum Tg [9]. In our cases, asymptomatic recurrent foci were detected by measuring the serum Tg level. Of note, whole body RAI scanning could also be used to detect recurrent foci after DTC surgery; however, a possibility of false positives exists for the kidneys because of other diseases, necessitating attention for evaluation [10, 11]. Although metastatic renal tumors require differentiation from primary RCC, CT and MRI do not have characteristic findings in metastatic renal tumor originating from thyroid cancer [7]. In our cases, a possibility of renal metastasis of thyroid cancer existed because the serum Tg level had increased. However, a case of thyroid cancer metastasized to RCC has been reported previously [12]. Whether the excision of distant metastasis contributes to the prognosis of thyroid cancer remains unclear. However, following consultation with urologists, we performed radical nephrectomy for diagnostic and therapeutic purposes in both cases. The efficacy of resection of distant metastases has been analyzed in various cancers. In colorectal cancer, hepatic resection is considered a standard treatment option for metastatic colorectal cancer and can result in 10-year survival rates of 20–26%, and potential cure. There is no established evidence in other cancers, but there are cases in which long-term prognosis is obtained by resection of oligometastasis [13]. Therefore, the resection of oligometastasis in thyroid cancer treatment might be considered a treatment option.

The pathology reports revealed characteristic features of DTC. A primary renal tumor that displays similar histology to FTC is known as thyroid-like follicular carcinoma of the kidney (TLFCK). TLFCK is an extremely rare histological variant of RCC; however, thyroid-specific immunohistochemical markers, such as TTF-1 and Tg, are negative [14]. In both cases, TTF-1 and Tg were positive by IHC, and it was feasible to diagnose renal metastasis of thyroid cancer.

RAI is used to treat DTCs that develop recurrence and distant metastasis. The prognosis of patients with the ^131^I uptake is better than those without the ^131^I uptake [15]. However, almost all studies reporting the efficacy of RAI comprised lung or bone metastasis cases, with no data demonstrating the efficacy of RAI for the treatment of renal metastases.

To date, radioiodine refractory DTC (RR-DTC) treatment options are very limited. However, sorafenib and lenvatinib, which are tyrosine kinase inhibitors (TKIs), have recently been approved and used to treat RR-DTCs [16, 17]. Although both drugs exhibit high efficacy, many patients are forced to discontinue because of adverse effects (AEs). In the DECISION trial, dose reductions were required in 64.3% of patients in the sorafenib group [16]. Similarly, in the SELECT trial, dose reductions were required in 67.8% of patients in the lenvatinib group and 90% of Japanese patients taking lenvatinib [17, 18]. Moreover, most patients in these trials had lung, lymph node, or bone metastasis, and the efficacy of TKI for renal metastasis remains unknown. In addition, AEs of TKI exhibit a possibility of substantially decreasing patients' quality of life. In our case, both patients were asymptomatic, and the efficacy of TKI was unknown for these disease conditions, thereby necessitating caution while introducing TKI. In contrast, surgery could result in definitive volume reduction. Overall, radical nephrectomy could be a reasonable treatment if there is no metastasis in other organs and it is not a case of bilateral kidney metastases [2, 7].

## 4. Conclusions

We reported cases of two patients with DTC who underwent nephrectomy with single metastasis to the kidneys. To date, the best treatment for renal metastasis of thyroid cancer remains debatable. However, this report suggests that nephrectomy for single renal metastasis could be considered a treatment option if patients' general condition is positive. Nevertheless, long-term follow-up is necessary to assess efficacy.
